# Unveiling 14 novel 2-hydroxy acid racemization and epimerization reactions in the lactate racemase superfamily

**DOI:** 10.1016/j.jbc.2024.108069

**Published:** 2024-12-10

**Authors:** Julian Urdiain-Arraiza, Amandine Vandenberghe, Gergana Dimitrova, Benoît Desguin

**Affiliations:** Louvain Institute of Biomolecular Science and Technology (LIBST), UCLouvain, Louvain-La-Neuve, Belgium

**Keywords:** NPN cofactor, hydroxy acids, racemase, epimerase, capillary electrophoresis

## Abstract

2-hydroxy acids are organic carboxylic acids ubiquitous in the living world and are important building blocks in organic synthesis. Recently, the lactate racemase (LarA) superfamily, a diverse superfamily of 2-hydroxy acid racemases and epimerases using the nickel-pincer nucleotide (NPN) cofactor, has been uncovered. In this study, we performed a taxonomic analysis of the LarA superfamily, showing the distribution of lactate racemase homologs (LarAHs) sequences across the three domains of life. Subsequently, we overexpressed and purified nine LarAHs and investigated their biochemical properties and substrate specificities. We show that LarAHs from the lactate racemases group are more promiscuous than previously thought, with some members showing high specificity towards glycerate or 2-hydroxybutyrate. In other phylogenetic groups, we identified a new malate racemase and 2-hydroxyglutarate racemase, as well as a new 2-gluconate epimerase from an eukaryotic organism. We show that some LarAHs are able to isomerize up to 16 different substrates, mostly 2-hydroxy acids with hydrophobic side chains, thereby identifying 14 novel 2-hydroxy acid racemization and epimerization reactions catalyzed by LarAHs. These include the racemization of glycerate, 2-hydroxybutyrate, 2,4-dihydroxybutyrate, 2-hydroxyvalerate, 2-hydroxycaproate, 2,3-dihydroxyisovalérate, 2-hydroxy-3,3-dimethylbutyrate, 3-(4-hydroxyphenyl)lactate, 2-hydroxy-4-phenylbutyrate, and 2-hydroxy-4-oxo-4-phenylbutyrate. Additionally, we observed the C2-epimerization of all 2,3-dihydroxybutyrate stereoisomers (4-deoxy-DL-threonate and 4-deoxy-DL-erythronate) and the C2-epimerization of D-arabinarate epimers. Finally, through comparative analysis of Alphafold structural predictions, we identified key residues likely involved in substrate specificity and predicted the function of half of the LarAHs from the LarA superfamily. In conclusion, this study widely expands the scope of substrates isomerized by NPN-dependent enzymes.

2-Hydroxy acid racemases (2-HARs) and epimerases (2-HAEs) are enzymes that play a crucial role in biochemical systems across a multitude of organisms by catalyzing the stereochemical inversion of the α-carbon of 2-hydroxy acids (2-HAs), a type of organic carboxylic acid ubiquitous in the living world.

2-HARs converts the two enantiomeric forms of 2-HAs with one chiral center (*e.g.* lactate racemase), whereas 2-HAEs act on 2-HAs with more than one chiral center by converting a stereoisomer into its epimer, which differs in the configuration of only one chiral center (*e.g.* D-gluconate epimerase). In both cases, the product of the reaction retains the same functional groups and connectivity than the substrate but shows different biological properties.

Moreover, 2-HAs are key metabolites and are involved in most metabolic pathways, including, but not limited to, the degradation of amino acids (1, 2, 3), the biosynthesis of cellular components (4), and the metabolism of sugars (5, 6). The racemization and epimerization of these compounds, catalyzed by 2-HARs and 2-HAEs, respectively, are critical for maintaining the equilibrium between different stereoisomers within the cell. 2-HARs and 2-HAEs are not only fundamental to cellular homeostasis but may also have implications for the synthesis of a broad range of biologically active molecules, including pharmaceuticals and agrochemicals (7, 8).

Recent discoveries have shed light on a superfamily of nickel-dependent 2-HARs and 2-HAEs, the LarA superfamily (9). The founding member of the LarA superfamily is *Lactiplantibacillus plantarum* lactate racemase (LarA1) which catalyzes the racemization of D- and L-lactate by using the Nickel-Pincer Nucleotide (NPN) cofactor (10, 11). The NPN cofactor is a substituted pyridinium mononucleotide that tricoordinates nickel with two sulfur atoms and one carbon atom, forming a stable Ni-C bond. NPN is bound to the catalytic site by a lysine residue (K184 in LarA1), an arginine residue (R75 in LarA1) interacting with the phosphate group, and a histidine residue (His200 in LarA1) providing the fourth ligand to the nickel (Fig. 1) (11). LarA1 and the NPN cofactor operate by a proton-coupled hydride-transfer (PCHT) mechanism during which the NPN transiently accepts the substrate-derived hydride. The PCHT mechanism of LarA1 start by either His108 or His174 (depending on the isomer of lactate) abstracting the proton from the C2-hydroxyl group of lactate. Concomitantly, the C2-hydrogen atom transfers as a hydride onto C4 of the pyridinium ring of the NPN cofactor, forming the intermediate pyruvate and a reduced NPN cofactor. The hydride then rapidly attacks the intermediate held at the active site, and either His108 or His174 (depending on the isomer of lactate) reprotonates the intermediate, resulting in lactate racemization (Fig. 1) (9, 12). The NPN cofactor is sequentially synthesized by the LarB, LarE, and LarC biosynthetic enzymes from the precursor nicotinic acid adenine dinucleotide through consecutive carboxylation/hydrolysis, sulfur insertion, and nickel insertion reactions, respectively (13, 14, 15, 16, 17, 18).

**Figure 1:**
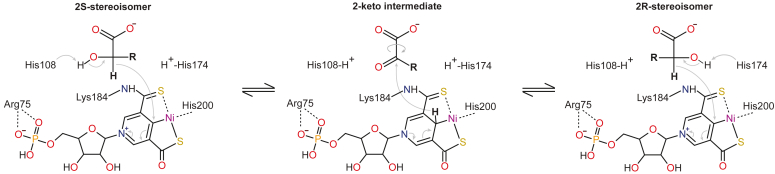
**NPN cofactor and proton-coupled hydride transfer reaction of LarAHs.** The residue numbers correspond to the residues of *L. plantarum* LarA1 (PDB code 5HUQ or 6C1W).

Interestingly, the genes encoding for the NPN-biosynthetic enzymes and LarA homologs (LarAHs) are widespread within eubacteria and archaea, and some are even found in unicellular eukaryotes (10). The high sequence diversity and genomic contexts of LarAHs within the LarA superfamily suggested that they may catalyze a wide range of enzymatic reactions. To date, several LarAHs have been shown to use the NPN cofactor and catalyze racemization or epimerization activities of the α-carbon of 2-HAs other than lactate. Two malate racemases (LarAH5/MAR1 and LarAH6/MAR2), a 2-hydroxyglutarate racemase (LarAH7/HGR), two D-gluconate two-epimerases (LarAH19/GntE1 and LarAH20/GntE2), a short-chain aliphatic 2-hydroxyacid racemase (LarAH2/SAR), and a 3-phenyllactate racemase (LarAH10/PLR) were identified among these LarAHs (9). LarA and the LarAHs likely share the same reaction mechanism in which the ɑ chiral carbon of a 2-HA is transiently oxidized generating an ɑ-keto acid intermediate (Fig. 1), while the active site residues unique to each family in the LarA superfamily allow the determination of the substrate specificity (9, 19). These latest discoveries likely represent just a fraction of the scope of racemization or epimerization reactions catalyzed by LarAHs. The specific enzymatic functions within the majority of phylogenetic families in the LarA superfamily remain largely unexplored and require further investigation.

In this study, we undertook a phylogenetic analysis and exploration of the LarA superfamily to uncover the scope of reactions catalyzed by different LarAHs. We have thoroughly characterized nine new LarAHs, predominantly from unexplored phylogenetic clusters. Among these enzymes, we have identified 14 previously unreported 2-HA racemization and epimerization reactions. Remarkably, certain LarAHs exhibit catalytic activity on over 16 distinct 2-HAs. These findings demonstrate the potential of the LarA superfamily for biotechnological and pharmaceutical applications in the synthesis of valuable 2-HA stereoisomers.

## Results

### Taxonomic analysis of the LarA superfamily

**Figure 2:**
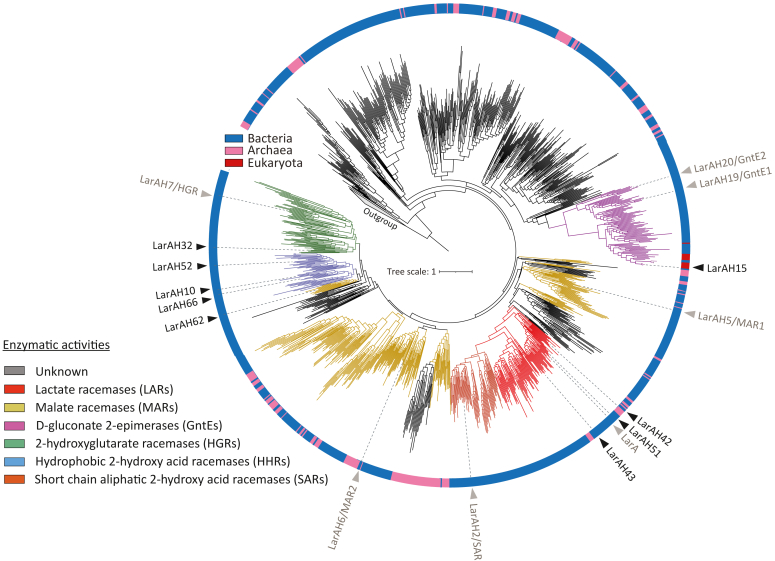
**Representative phylogenetic tree of the LarA superfamily.** The tree includes 1212 representative LarAHs sequences from DUF2088 and 10 sequences from DUF362 used as an outgroup. All LarAHs mentioned in this report are indicated as “LarAHX” at their respective phylogenetic position. LarAHs characterized in this work are shown in *black*, previously characterized LarAHs are shown in *gray*. The colors of the phylogenetic groups correspond to the different reactions identified in these groups. The taxonomic group each sequence belongs to is labeled by a color strip at the circumference and the color code can be cross-referenced with Fig. S1. LarA1 is from *Lactiplantibacillus plantarum*; LarAH2/SAR is from *Isosphaera pallida*; LarAH5/MAR1 is from *Desulfitobacterium hafniense*; LarAH6/MAR2 is from *Thermoanaerobacterium thermosaccharolyticum*; LarAH7/HGR is from *Deferribacter desulfuricans*; LarAH19/GntE1 is from *Corynebacterium glutamicum*; LarAH20/GntE2 is from *Thermotoga maritima*; LarAH10 is from *Megasphaera elsdenii*; LarAH15 is from *Phytophthora nicotianae*; LarAH32 is from *Geobacter metallireducen*s; LarAH42 and LarAH43 are from *Enterocloster asparagiformis*; LarAH51 and LarAH52 are from *Clostridium pasteurianum*; LarAH62 and LarAH66 are from *Sporomusa termitida*.

The LarA superfamily, also referred to as the DUF2088 superfamily due to its previously unknown enzyme functions, represents a highly diverse group of enzymes (20). This superfamily includes LarA-like domains, corresponding to the Interpro entry IPR048068 (21), which encompasses currently a total of 12,441 LarAHs found across 3804 species, mostly bacteria (Fig. S1*A*). Among bacteria, the majority of LarAHs are currently observed in taxa such as *Bacillota* (*Firmicutes*), *Actinomycetota*, *Pseudomonadota* (*Proteobacteria*) and *Planctomycetota* (Fig. S1*B*). The phylogenetic tree built from the alignment of 1212 representative LarAHs sequences shows the diversity of sequences in the LarA superfamily (Fig. 2), suggesting that many racemization or epimerization reactions of 2-HAs could still be discovered.

**Figure 3:**
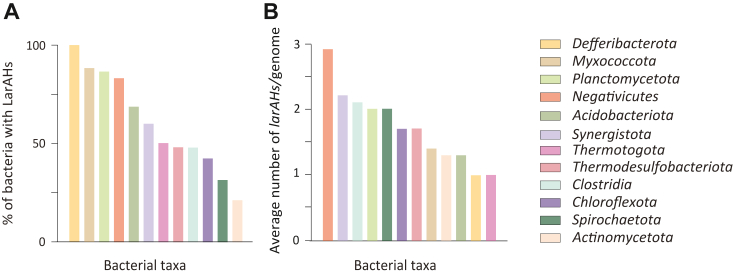
**Bacterial taxa with the highest prevalence of LarAHs.***A*, bacterial taxa with the highest percentage of bacteria with LarAHs. *B*, bacterial taxa with the highest average number of *larAHs* per genome. All bacterial taxa represented are phyla, with the exception of *Clostridia* and *Negativicutes,* which are classes of bacteria within the phylum *Bacillota* (*Firmicutes*). Data assembled from the database of Clusters of Orthologous Genes (COGs) entry COG3875 on the National Center for Biotechnology Information (NCBI) obtained on September 23, 2024.

Using the Clusters of Orthologous Genes (COGs) entry COG3875, the prevalence of *larAH* sequences across archaea and bacteria was analyzed, revealing distinct distribution patterns. In archaea, 29.5% of species contain at least one *larAH* sequence, with the highest prevalence observed in *Asgardarchaeota* (87.5%, Table S1). In bacteria, 16.4% of species harbor at least one *larAH* sequence, with *Deferribacterota*, *Myxococcota*, *Planctomycetota*, and *Negativicutes* showing the highest abundance (80% to 100%, Fig. 3*A* & Table S1). Most species possess a single *larAH* sequence, although species from several bacterial taxa, such as *Negativicutes*, *Synergistota*, *Clostridia*, *Planctomycetota* and *Spirochaetota*, possess an average of two or more *larAHs* per genome (Fig. 3*B* & Table S1). Notably, taxa with the highest prevalence of LarAHs are predominantly anaerobic, although a few, including *Actinomycetota* (*e.g.*, *Corynebacterium glutamicum*), *Planctomycetota* (*e.g.*, *Planctomyces limnophilus*), and *Acidobacteriota* (*e.g.*, *Luteitalea pratensis*), contain aerobic species with LarAHs (Fig. 3). These findings highlight the diverse distribution of *larAH* sequences among different taxa, suggesting a high potential ecological significance.

### Biochemical characterization of nine selected LarAHs

To gain further insight into the LarA superfamily, we selected nine *larAH* genes from five distinct bacteria and one eukaryote (Fig. 2). Subsequently, we engineered vectors to generate the respective C-terminal Strep-Tag fusion proteins for overexpression and purification in *Escherichia coli* cells. After purification, the nine LarAHs were obtained in soluble form and their identity was confirmed by electrospray ionization quadrupole time-of-flight mass spectrometry (ESI-Q-TOF-MS) analysis (Figs. S2 and S3, Table S2).

**Figure 4:**
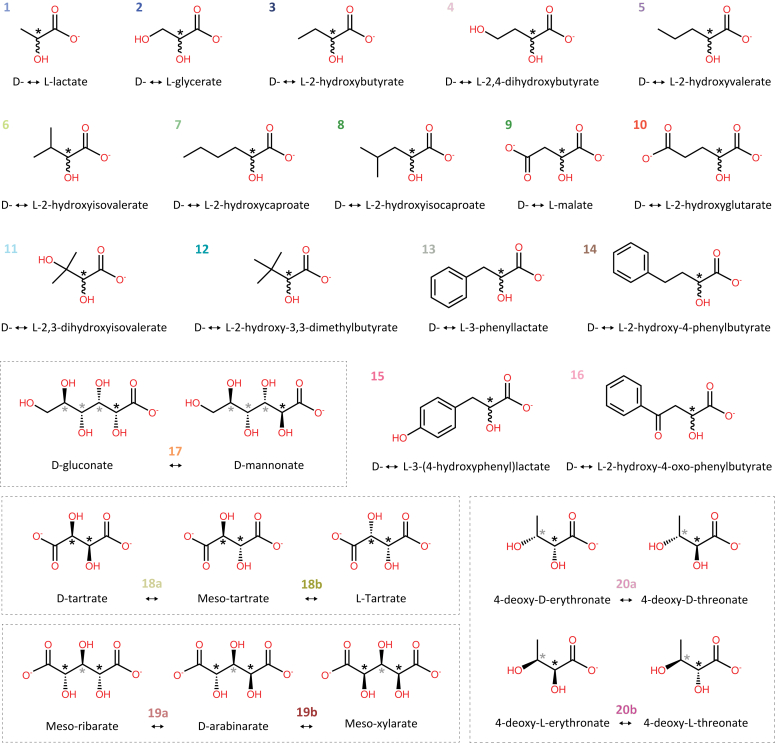
**Chemical structure of substrates racemized and epimerized by LarAHs.** For racemization reactions, the chiral carbons of enantiomers are depicted achiral for simplification. For C2-epimerization reactions, both epimers are depicted, with diastereomers of the same molecule boxed together. *Black* asterisks indicate stereoinversion sites, *grey* asterisks indicate other stereocenters in the molecule.

The purified LarAHs were then supplemented with *in vitro* synthesized NPN and subjected to activity assays in the presence of a range of 2-HAs (Fig. 4). These assays were conducted through either coupling to another enzymatic reaction for spectrophotometric measurement or *via* chiral capillary electrophoresis (CE) (21).

For a comprehensive biochemical characterization of each LarAH, we experimentally determined their optimal pH and temperature conditions, as well as their kinetic parameters for one representative substrate (*k*_cat_, *K*_M_ and *k*_cat_*/K*_M_) (Table 1 & Figs. S4–S6). Temperature and pH profiles of the nine investigated LarAHs showed that the majority of LarAHs exhibited a pH optimum around pH seven and a temperature optimum around 40 °C, which is expected for enzymes from mesophilic organisms. Kinetic studies showed their affinity for their substrate was generally higher than the affinity of LarA1 for lactate, yet their *k*_*cat*_ was generally lower, resulting in similar catalytic efficiencies for most LarAHs. However, some LarAHs showed significantly lower catalytic efficiencies (Table 1). Kinetic parameters of the NPN-supplemented enzymes were determined by assuming 100% loading, given that an excess of NPN was added to the apoprotein; however, the extent of NPN loading could not be established. As a result, the values of *k*_cat_ and *k*_cat_/*K*_M_ are probably underestimated.

**Table 1:** Kinetic parameters of LarA1 and of the nine investigated LarAHs

Enzyme	Substrate	pH opt^a^	Temp opt^a^ (°C)	*K*_M_ (mM)^b^	*k*_cat_ (s^−1^)^b^	*k*_cat_*/K*_M_ (M^−1^ s^−1^)
LarA1	D-lactate	6	35–40	11 ± 4	1300 ± 150	(12 ± 6) × 10^4^
	L-lactate			46 ± 20	4750 ± 500	(10 ± 5) × 10^4^
LarAH42	D-lactate	7	35–40	4.4 ± 0.5	1.1 ± 0.2	(22 ± 5) × 10^1^
	L-lactate			6.9 ± 1.2	1.4 ± 0.3	(20 ± 5) × 10^1^
LarAH43	D-lactate	7–7.5	40–50	0.45 ± 0.04	30 ± 3	(66 ± 7) × 10^3^
	L-lactate			0.51 ± 0.08	42 ± 5	(83 ± 9) × 10^3^
LarAH51	D-lactate	6	40–45	0.33 ± 0.02	40 ± 6	(118 ± 18) × 10^3^
	L-lactate			0.49 ± 0.02	48 ± 4	(98 ± 9) × 10^3^
LarAH32	L-2-hydroxyglutarate	7–8	40–45	0.27 ± 0.05	4.1 ± 0.8	(15 ± 4) × 10^3^
LarAH10	D-mannonate	7.5–8	30–35	3.8 ± 0.8	0.7 ± 0.5	(17 ± 4) × 10^1^
LarAH52	D-2-hydroxy-4-oxo-phenylbutyrate	5.5–6.5	30–35	44 ± 8	510 ± 60	(12 ± 2) × 10^3^
LarAH15	D-mannonate	6.5–7	40–50	9.4 ± 1.5	111 ± 12	(12 ± 3) × 10^3^
LarAH62	D-malate	6–6.5	40	0.21 ± 0.02	138 ± 8	(63 ± 7) × 10^4^
LarAH66	D-2-hydroxybutyrate	8	40	25 ± 4	0.92 ± 0.11	37 ± 5
	D-lactate			28 ± 6	0.67 ± 0.12	24 ± 7

a opt: optimal pH range or temperature (temp), where activity was > 90% of the maximum activity.

b ± 95% confidence interval based on non-linear regression using the Michaelis–Menten equation.

**Figure 5:**
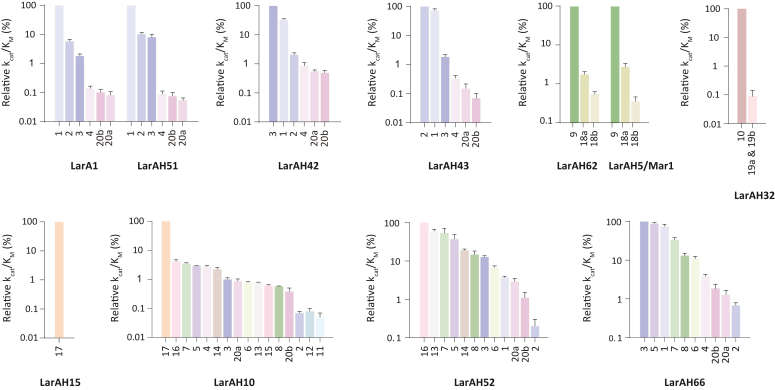
**Relative catalytic efficiencies of LarA1 and of the nine investigated LarAHs.** The catalytic efficiency for the preferred substrate was set to 100%. The error bars represent the standard error (n = 3).

We then determined the relative catalytic efficiencies for all substrates identified for each LarAH (Fig. 5). This analysis allowed us to identify the preferred substrates of each LarAH and evaluate their specificity for each other substrate. The *k*_cat_/*K*_M_ values of all substrates of each LarAH were calculated using the experimentally determined *k*_cat_/*K*_M_ for one substrate and the relative catalytic efficiencies for all substrates (Table 2).

**Table 2:** Catalytic efficiencies of LarA1 and of the nine investigated LarAHs

Reactions		*k*_cat_*/K*_M_ (M^−1^ s^−1^)			
		LarA1	LarAH51	LarAH43	LarAH42
1	L- ↔ D-lactate	**(10 ± 5) × 10**^**4**^	**(98 ± 9) × 10**^**3**^	**(83 ± 9) × 10**^**3**^	**(20 ± 5) × 10**^**1**^
2	L- ↔ D-glycerate	(6 ± 3) × 10^3^	(102 ± 12) × 10^2^	(114 ± 20) × 10^3^	12 ± 3
3	L- ↔ D-2-hydroxybutyrate	(2 ± 1) × 10^3^	(80 ± 8) × 10^2^	(21 ± 5) × 10^2^	(61 ± 15) × 10^1^
4	L- ↔ D-2,4-dihydroxybutyrate	(14 ± 7) × 10^1^	(9 ± 3) × 10^1^	(4 ± 1) × 10^2^	5 ± 2
20a	4-deoxy-D-threonate ↔ 4-deoxy-D-erythronate	(8 ± 4) × 10^1^	(5 ± 2) × 10^1^	(17 ± 8) × 10^1^	3 ± 1
20b	4-deoxy-L-threonate ↔ 4-deoxy-L-erythronate	(10 ± 5) × 10^1^	(7 ± 2) × 10^1^	(8 ± 3) × 10^1^	3 ± 1
		LarAH10	LarAH52	LarAH66	LarAH15
1	L- ↔ D-lactate	-	(34 ± 7) × 10^1^	**28 ± 6**	-
2	L- ↔ D-glycerate	0.12 ± 0.03	18 ± 9	0.25 ± 0.06	-
3	L- ↔ D-2-hydroxybutyrate	1.7 ± 0.5	(12 ± 3) × 10^2^	**37 ± 5**	-
4	L- ↔ D-2,4-dihydroxybutyrate	5.3 ± 1.6	-	1.40 ± 0.32	-
5	L- ↔ D-2-hydroxyvalérate	5.1 ± 1.2	(34 ± 12) × 10^2^	33.0 ± 6.5	-
6	L- ↔ D-2-hydroxyisovalérate	1.4 ± 0.3	(62 ± 14) × 10^1^	4.0 ± 1.1	-
7	L- ↔ D-2-hydroxycaproate	6.1 ± 1.4	(49 ± 17) × 10^2^	12.0 ± 2.9	-
8	L- ↔ D-2-hydroxyisocaproate	1 ± 0.3	(14 ± 4) × 10^2^	4.8 ± 1.2	-
11	L- ↔ D-2,3-dihydroxyisovalérate	0.10 ± 0.04	-	-	-
12	L- ↔ D-2-hydroxy-3,3-dimethylbutyrate	0.13 ± 0.05	-	-	-
13	L- ↔ D-3-phenyllactate	1.3 ± 0.3	(55 ± 13) × 10^2^	-	-
14	L- ↔ D-2-hydroxy-4-phenylbutyric acid	3.9 ± 1.1	(17 ± 4) × 10^2^	-	-
15	L- ↔ D-4-hydroxyphenyllactate	1.0 ± 0.3	-	-	-
16	L- ↔ D-2-hydroxy-4-oxo-4-phenylbutyrate	7.5 ± 1.9	**(92 ± 18) × 10**^**2**^	-	-
17	D-gluconate ↔ D-mannonate	**(17 ± 4) × 10**^**1**^	-	-	**(12 ± 3) × 10**^**3**^
20a	4-deoxy-D-threonate ↔ 4-deoxy-D-erythronate	1.2 ± 0.3	(10 ± 4) × 10^1^	0.69 ± 0.19	-
20b	4-deoxy-L-threonate ↔ 4-deoxy-L-erythronate	1.1 ± 0.3	(27 ± 8) × 10^1^	0.25 ± 0.06	-
		LarAH27 [9]	LarAH62	LarAH32	
9	L- ↔ D-malate	**(39 ± 7) × 10**^**1**^	**(63 ± 7) × 10**^**4**^	-	
18b	L- ↔ Meso-tartrate	2 ± 1	(35 ± 8) × 10^2^	-	
18a	D- ↔ Meso-tartrate	14 ± 4	(11 ± 2) × 10^3^	-	
10	L- ↔ D-2-hydroxyglutarate	-	-	**(15 ± 4) × 10**^**3**^	
19a-b	Meso-ribarate ↔ D-arabinarate ↔ Meso-xylarate	-	-	13 ± 6	

For each LarAH, the *k*_cat_*/K*_M_ value of the substrate for which the kinetic parameters were obtained experimentally is in bold (Fig. S6 & Table 1). Additional *k*_cat_/*K*_M_ values were calculated using the experimentally determined *k*_cat_/*K*_M_ and the relative *k*_cat_/*K*_M_ values. “-”: no activity.

This study revealed that lactate racemases (LARs) exhibit a greater degree of promiscuity than previously assumed. All LARs were found to be capable of catalyzing the racemization of several other small 2-HAs, including glycerate and 2-hydroxybutyrate. In some instances, their catalytic efficiency was even higher than that of lactate, which is traditionally regarded as the major substrate of LARs (Fig. 5 & Table 2). We also identified an additional MAR, an additional HGR and an additional GntE, which all showed a very narrow substrate spectrum (Fig. 5 & Table 2). Finally, we discovered a new family of LarAHs, comprising LarAH10, LarAH52 and LarAH66 which were found to act on up to 16 different substrates, predominantly 2-HAs with hydrophobic side chains (Fig. 5 & Table 2). One member of this group had already been identified previously, but only one substrate had been identified at the time: 3-phenyllactate (9). Given that these LarAHs are capable of racemizing a diverse range of 2-HAs, we have chosen to refer to these new LarAHs as hydrophobic 2-HA racemases (HHRs) rather than 3-phenyllactate racemases. Most of the discovered reactions have not been previously reported, resulting in the identification of 14 novel 2-HA racemization and epimerization reactions (Fig. 5 & Table 2).

### Structural model analysis of nine selected LarAHs and exploration of the phylogenetic tree

**Figure 6:**
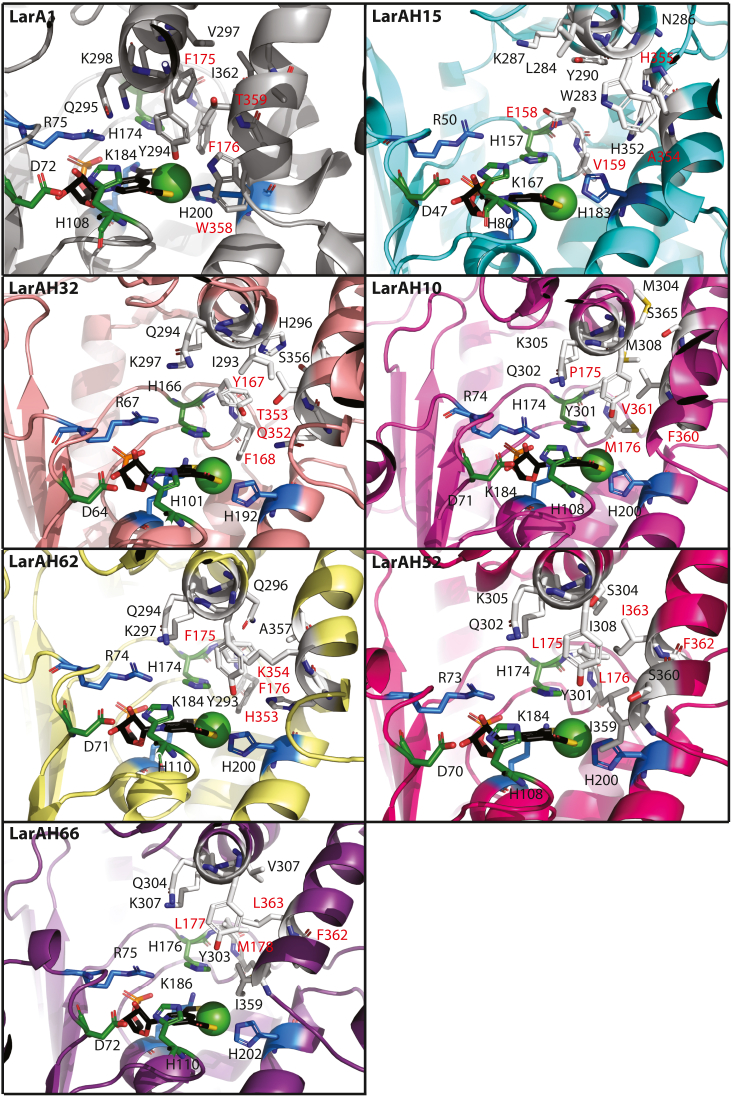
**Structure of LarA1 (PDB code**5HUQ) **and structural models of six LarAHs.** The catalytic residues are shown with their carbons in *green*, the residues important for NPN binding are shown with their carbon in *blue*, other resides probably important for substrate specificity are shown with their carbons in *white*. NPN carbons are in *black*. Oxygen atoms are in *red*, nitrogen atoms are in *blue*, sulfur atoms are in *yellow*, phosphate atoms are in *orange*, and nickel ion is shown as a *green* ball. The residues corresponding to F175, F176, W358, and T359 of LarA1 are labelled in *red*.

The structural models of the nine LarAHs were retrieved from the AlphaFold database (22, 23). These models were aligned with the LarA1 structure (PDB code 5HUQ) using Pymol in order to deduce the position of the NPN cofactor. The catalytic histidines, corresponding to His108 and His174 in LarA1, are shown in green, the residues important for NPN binding, corresponding to R75, K184, and His200 in LarA1, are shown in blue, and other resides probably important for substrate specificity are in white (Figs. 6 & S7).

With regard to the group comprising LARs, very few differences are observed in the catalytic site (Fig. S7). The most significant difference, which may account for the observed differences in substrate specificities, is the residue corresponding to T359 in LarA1, which is equivalent to E357 in LarAH51, Q359 in LarAH43, and C357 in LarAH42. As the glutamine residue is more hydrophilic than threonine residues and is a good hydrogen bond acceptor, this could explain the high specificity of LarAH43 for glycerate (Fig. S7). The catalytic pocket of LarAH15 and of LarAH62 is similar to that of previously identified GntEs and MARs, respectively (Fig. 6) (9).

The HGR LarAH32 showed a catalytic site comprising several hydrophilic residues, including a glutamine at position 352 and a threonine at position 353 (Fig. 6), which were also present in the sequence of the previously identified HGR (9). The newly identified group of HHRs, comprising LarAH10, LarAH52 and LarAH66, exhibit a high prevalence of hydrophobic residues within their catalytic site. LarAH10 exhibits three methionine residues in its catalytic site (Fig. 6), which are flexible and can likely adopt different conformations depending on the substrate. This explains the wide range of possible substrates on which LarAH10 can act (Fig. 5 & Table 2). In contrast, LarAH52, is more specific for 2-HAs with bulky side chains, whereas LarAH66 acts preferentially on 2-HAs with small side chains (Fig. 5 & Table 2). Nevertheless, this difference in specificity is not readily apparent from the structural models of the catalytic sites of LarAH52 and LarAH66 (Fig. 6).

Based on the results obtained for the new LarAHs (Fig. 5 & Table 2) and on their structural models (Fig. 6), we assigned probable functions to as many unknown LarAHs as possible on the phylogenetic tree of the LarA superfamily (Fig. 2). The residues equivalent to F175, F176, W358, and T359 in LarA1 were previously identified as important for substrate selectivity (9, 19), so we focused on these four residues. LARs usually exhibit F, F, W, and E/Q at these positions. GntEs usually exhibit E, V, A, and H at these positions. HGRs usually exhibit Y, F, H/Q and T at these positions. MARs usually exhibit H/Q and K at the positions corresponding to W358 and T359 in LarA1, with the first two positions being more variable. In MARs, the positive charge of the K, at the position corresponding to T359 in LarA1, probably stabilizes the negative charge of the second carboxylate of malate by forming a hydrogen bond. In contrast, HHRs appear to exhibit a markedly lower degree of residue conservation at these positions. However, a general observation can be made regarding HHRs: the presence of a F/Y at the position corresponding to W358 in LarA1, which leaves more space for the wide range of HHR substrates to fit in (Fig. 6). Based on these observations, we have predicted the function of more than half the LarAHs of the LarA superfamily (Fig. 2).

## Discussion

### Lactate racemases (LARs)

Our exploration of the LarA superfamily started with an investigation of the phylogenetic subgroups surrounding LarA1, aiming to deepen our understanding of this phylogenetic branch and explore the potential catalytic versatility beyond lactate racemization. We characterized the substrate specificity of three novel LARs belonging to distinct subgroups of the branch: LarAH42 and LarAH43 from *Enterocloster asparagiformis*, and LarAH51 from *Clostridium pasteurianum* (Fig. 5 & Table 2). Our findings highlight the broader catalytic potential of LARs, demonstrating their ability to accommodate a diverse range of very short-chain 2-HAs, in addition to lactate. Notably, despite originating from the same organism, LarAH42 and LarAH43 exhibit distinct catalytic efficiencies, suggesting specialized *in vivo* roles: LarAH42 appears to be more specialized in the racemization of 2-hydroxybutyrate, while LarAH43 is more active on lactate and glycerate (Fig. 5 & Table 2). The physiological significance of these activities remains to be determined through *in vivo* investigations.

### Hydrophobic 2-hydroxyacid racemases (HHRs)

Our findings also revealed that LarAHs from various bacterial phylogenetic groups within the LarA superfamily phylogenetic tree catalyze the racemization of a wide range of 2-HAs with hydrophobic side chains. For instance, LarAH52 from *C*. *pasteurianum* catalyzes the isomerization of 12 compounds, with a preference for 2-HAs with a side chain containing an aromatic ring, notably 2-hydroxy-4-oxo-4-phenylbutyrate and 3-phenyllactate (Fig. 5 & Table 2). However, its low affinity for these substrates suggests that the preferred substrate we identified may not be the native substrate of the enzyme (Table 1). One hypothesis is that this enzyme may racemize indole-3-lactic acid, a product of tryptophan metabolism that regulates intestinal homeostasis through epithelium–macrophage interactions (24), which unfortunately is not readily available in its pure enantiomeric form. Similarly, LarAH10 from *Megasphaera elsdenii* exhibits isomerization activity on 16 different HAs, mostly 2-HAs with hydrophobic side chains. To our surprise, LarAH10 also showed the ability to catalyze the C2-epimerization of D-gluconate/D-mannonate, which had not been detected previously (10), probably due to the low *k*_*cat*_ of this enzyme (Table 1). Like LarAH52, LarAH10 displays a higher *k*_cat_/*K*_M_ for bulky 2-HAs. LarAH66, on the other hand, shows specific activity towards small 2-HAs, such as 2-hydroxybutyrate and 2-hydroxyvalerate, indicating a smaller catalytic site (Fig. 5 & Table 2). These differences cannot be inferred form the observation of the structural models, which shows the limits of these models (Fig. 6). Furthermore, LarAH10, LarAH52 and LarAH66 prefer substrates with linear side chains, compared to branched side chains. For example, these three LarAHs display a higher *k*_cat_/*K*_M_ for 2-hydroxyvalerate compared to 2-hydroxyisovalerate, and for 2-hydroxycaproate compared to 2-hydroxyisocaproate (Fig. 5 & Table 2). Most of the new reactions discovered were observed in this group of HHRs, nevertheless, we believe that only one substrate is the native substrate *in vivo* for each of these enzymes. For LarAH10, this seems to be D-gluconate, which is surprising as this LarAH is not similar to typical GntE, this might be a case of convergent evolution. For LarAH52 and LarAH66, we might not have identified the true substrate and the observed reactions might all be promiscuous reactions, which would explain the high *K*m values observed for these two enzymes (Table 1).

### Malate racemases (MARs)

MARs, found in both bacteria and archaea, form the largest group of enzymes within the LarA superfamily and are observed in different phylogenetic groups, emphasizing the importance of MAR activity in microorganisms (Fig. 2). In addition to the two enzymes which have been previously identified as MARs (9), we identified LarAH62 from *Sporomusa termitida*, which exhibits a main racemization activity on malate and a promiscuous C2-epimerization activity on tartrate, similar to what was observed for LarAH6/MAR2 (19) and for LarAH5/MAR1 (Fig. 5 & Table 2). Both substrates share a similar 4-carbon backbone with two carboxylates.

### D-gluconate two-epimerases (GntEs)

GntEs are found exclusively in bacteria, but a few also come from unicellular eukaryotes. In addition to the two previously identified GntEs (10), we identified LarAH15 as a GntE. Notably, LarAH15 is from *Phytophthora nicotianae*, a model soilborne oomycete pathogen known to infect a broad spectrum of host plants (25), making it the first eukaryotic LarAH enzyme with confirmed activity. Interestingly, all LarAHs from eukaryotic organisms are part of the GntE phylogenetic branch, indicating a likely significant role for D-gluconate C2-epimerization in some unicellular eukaryotes. These GntEs could be fungicide targets for managing *Phytophthora* diseases of crops.

### 2-Hydroxyglutarate racemases (HGRs)

HGRs are found exclusively in bacteria. In addition to the previously identified HGR (9), we identified LarAH32 from *Geobacter metallireducens*, which exhibits a main racemization activity on 2-hydroxyglutarate and a promiscuous C2-epimerization activity on D-arabinarate. Both substrates share a similar 5-carbon backbone with two carboxylates.

## Conclusion

In conclusion, we significantly expanded the scope of NPN-dependent reactions catalyzed by members of the LarA superfamily. Through a comprehensive approach involving taxonomic analysis, biochemical characterization, and structural modeling, we have identified and characterized the substrate specificities of 8 novel LarAHs. We identified three additional LARs, one additional MAR, one additional HGR, one additional GntE, and two additional HHRs. We showed that HHRs exhibit broad activity on a wide range of 2-HAs, with some capable of catalyzing up to 16 distinct isomerization reactions. Furthermore, the prevalence of LARs, MARs, GntEs, HHRs, and HGRs within the LarA superfamily suggests the metabolic importance of these enzymes across diverse organisms. Taken together, our findings demonstrate the remarkable adaptability and importance of NPN-dependent enzymes for the catalysis of isomerization reactions in bacteria and archaea. The study of LarAHs is a completely new field of research that could have many implications in bacterial and archaeal physiology and lead to potential medical or industrial applications, *e.g.*, for the optimization of probiotics, for the production of rare isomers of 2-HAs, or as fungicide targets in oomycete pathogens. Looking ahead, further exploration of LarAHs promises to uncover additional enzymatic functions and metabolic pathways, facilitating the development of novel biocatalysts and potential biotechnological applications.

## Experimental procedures

### Biological materials and growth conditions

Bacterial strains and plasmids used in the present study are listed in Table S3. All plasmid constructions were performed in *E. coli* DH10B. *E. coli* DH10B cells were grown with agitation at 37 °C in lysogeny broth with ampicillin (200 mg·L − 1) or erythromycin (200 mg·L − 1), when required. When expressing pBADHisA derivatives, induction was initiated by addition of L-arabinose (0.1%) and cells were grown at 30 °C for 4 h with agitation.

### DNA techniques

DNA was introduced into *E. coli* cells by electrotransformation (26). PCR amplifications used Q5 high-fidelity DNA polymerase (New England Biolabs). The primers used in this study were purchased from Eurogentec and are listed in Table S4. Synthetic genes for LarAH15, LarAH10 and LarAH32 sequences were purchased from Biomatik, digested with NcoI and NheI, and cloned into a similarly digested pGIR076 (16). Genes encoding LarAH42 and LarAH43 were amplified from the genomic DNA of *E. asparagiformis*, digested (PciI and NheI), and cloned into digested (NcoI and NheI) pGIR076. Genes encoding LarAH51 and LarAH52 were amplified from the genomic DNA of *C. pasteurianum*, digested (PciI and NheI), and cloned into digested (NcoI and NheI) pGIR076. Genes encoding LarAH62 and LarAH66 were amplified from the genomic DNA of *S. termitida*, digested (PciI and NheI for LaraH62, NcoI and NheI for LarAH66), and cloned into digested (NcoI and NheI) pGIR076. *E. asparagiformis* (DSM 15981), *C. pasteurianum* (DSM 525), and *S. termitida* (DSM 4440) genomes were purchased from DSMZ-German Collection of Microorganisms and Cell Cultures. In all cases, the sequence of the expression cassettes was verified by sequencing. Strains and primers used are listed in Tables S3 and S4, respectively.

### Protein purification and *in vitro* NPN biosynthesis

*In vitro* biosynthesis of NPN was performed with purified LarB, LarE, and LarC as previously described (15, 27). *E. coli* cells were induced to express LarAHs when the OD600 reached 0.4 with 0.1% L-arabinose. After 4 h, the culture was cooled to 4 °C, and the cells were harvested by centrifugation at 6000*g* for 10 min. The pellet was stored at −80 °C until further use. The cell pellet was thawed and resuspended in 20 ml of lysis buffer containing 100 mM Tris-HCl (pH 7.5), 300 mM NaCl and 1 mg/ml lysozyme. The cell suspension was incubated at 4 °C for 30 min and sonicated 4 times for 1 min with one 1-min rest on ice between each sonication. The supernatant was separated by centrifuging the lysate at 20,000*g* for 30 min at 4 °C. Clarified supernatant was loaded onto 2 ml of Strep-Tactin XT Superflow (IBA) resin equilibrated with 10 ml of wash buffer (W) composed of 100 mM Tris-HCl (pH 7.5), 300 mM NaCl. After loading, the resin was washed with 20 ml of W followed by elution with 15 ml of W containing 50 mM D-biotin (Sigma-Aldrich). The eluted proteins were ultracentrifuged using Amicon Ultra—15 ml MWCO 10 kDa filter units to increase purified protein concentration. The identities of each purified LarAH protein were confirmed by electrospray ionization–time-of-flight mass spectrometry analysis. The protein concentrations were estimated with the Bradford assay.

### Chemicals and reagents

All chemicals were either of analytical grade or reagent grade. D-lactic acid (sodium salt), L-lactic acid (sodium salt), D-glyceric acid (sodium salt), L-glyceric acid (sodium salt), D-malic acid, L-malic acid, D-2-hydroxybutyric acid, L-2-hydroxybutyric acid, DL-2-hydroxycaproic acid, DL-2-hydroxyisocaproic acid, L-2-hydroxyisocaproic acid, D-2-hydroxyisovaleric acid, L-2-hydroxyisovaleric acid, D-2,3-dihydroxyisovaleric acid (sodium salt), L-2,3-dihydroxyisovaleric acid (sodium salt), D-2-hydroxyglutaric acid (disodium salt), L-2-hydroxyglutaric acid (disodium salt), D-3-phenyllactic acid, L-3-phenyllactic acid, D-2-hydroxy-4-phenylbutyric acid, DL-mandelic acid, D-mandelic acid, D-gluconic acid (sodium salt), benzoic acid and L-histidine were purchased from Sigma-Aldrich. L-2-hydroxyvaleric acid, L-2-hydroxycaproic acid, and L-2-hydroxy-4-phenylbutyric acid, were purchased from Biosynth. DL-2-hydroxyvaleric acid was purchased from Acros Organics. 4-Deoxy-D-erythronic acid (sodium salt hydrate), 4-Deoxy-L-erythronic acid (sodium salt hydrate), 4-Deoxy-D-threonic acid (sodium salt hydrate), 4-Deoxy-L-threonic acid (sodium salt hydrate) were purchased from Supelco. Vancomycin was purchased from Thermo Fisher Scientific.

### Enzymatic assays

The general protocol for assaying LarAHs was as follows if not stated otherwise: 1 μM of *in vitro* synthesized NPN (final concentration), 30 mM substrate, 0.2 μM of purified LarAH (final concentration), 100 mM Tris–HCl buffer pH 8, in 50 μl final volume for 20 min at 30 °C.

For the discovery of LarAH activities, reactions were performed with 5 μM of *in vitro* synthesized NPN and 2 μM of purified LarAH for 12 h.

For the determination of temperature profiles, LarAHs were incubated at the indicated temperatures.

For the determination of pH profiles, LarAHs were incubated with acetic acid buffer (pH four to pH 5), potassium phosphate buffer (pH six to pH 8) or borate buffer (pH nine to pH 10).

For *K*_M_ measurements, reactions were performed with variable concentrations of substrate, 1 μM of *in vitro* synthesized NPN, 0.1 μM of purified LarAH, 100 mM Tris–HCl buffer pH 8, in 50 μl final volume for 20 min at 30 °C.

For *k*_cat_ measurements, reactions were performed with the substrate concentration corresponding to the V_max_ of LarAHs for 5 min at the optimal pH and temperature of LarAHs.

All reactions were then stopped by incubation at 90 °C for 10 min and the products were assayed spectrophotometrically or by capillary electrophoresis (28). D-lactate, L-lactate, D-malate, D-2-hydroxyglutarate racemization reactions and D-mannonate epimerization reactions were assayed spectrophotometrically at 340 nm with an Infinite 200 PRO plate reader (Tecan) using Megazyme kits. D-Lactate and L-lactate racemization reactions were assayed using the D-/L-Lactic Acid Assay Kit from Megazyme. D-malate racemization reactions were assayed using the L-Malic Acid Assay Kit from Megazyme. D-gluconate/D-mannonate epimerization reactions were assayed using the D-Gluconic Acid Assay Kit from Megazyme. L-2-hydroxyglutarate racemization reactions were assayed using *Acidaminococcus fermentans* D-hydroxyglutarate dehydrogenase (HGDH) (29), as previously described (9). All other racemization and epimerization reactions shown in Figure 4 were assayed by capillary electrophoresis (28).

### Capillary electrophoresis

Enantiomers and epimers of HAs have been separated and detected using a versatile capillary electrophoresis method allowing the direct chiral separation of aliphatic and aromatic 2-HAs and poly-HAs (28). Reaction mixtures were loaded onto a polyacrylamide-coated capillary of 54/46 cm total/effective length with an internal diameter of 50 μm from Agilent, and run on a Capel 105 M from Lumex Instrument at 20 °C using −25 kV. Using a modified partial filling-counter current method with indirect UV detection, high resolution was achieved with vancomycin as a chiral selector added to the background electrolyte composed of 10 mM of benzoic acid/L-histidine at pH 5. Products were detected at 230 nm.

### Peptide separation using nanoUPLC

Proteolysis was performed with 1 μg of sequencing grade trypsin (Promega) and allowed to continue overnight at 37 °C. Each sample was dried under vacuum with Savant Speed Vac Concentrator. Digested proteins were dissolved in 20 μl of 0.1% (v/v) formic acid and 2% (v/v) acetonitrile (ACN). Peptide mixture was separated by reverse phase chromatography on a NanoACQUITY UPLC MClass system (Waters) working with MassLynx V4.1 (Waters) software. 200ng of digested proteins were injected on a trap C18, 100Å 5 μm, 180 μm × 20 mm column (Waters) and desalted using isocratic conditions with at a flow rate of 15 μl/min using a 99% formic acid and 1% (v/v) ACN buffer for 3 min. Peptide mixture was subjected to reverse phase chromatography on a C18, 100Å 1.8 μm, 75 μm × 150 mm column (Waters) PepMap for 130 min at 35 °C at a flow rate of 300 nl/min using a two part linear gradient from 1% (v/v) ACN, 0.1% formic acid to 35% (v/v)) ACN, 0.1% formic acid for 90 min and from 35% (v/v) ACN, 0.1% formic acid to 85% (v/v)) ACN, 0.1% formic acid for 10 min. The column was re-equilibrated at initial conditions after washing 30 min at 85% (v/v)) ACN, 0.1% formic acid at a flow rate of 300 nl/min. For online LC-MS analysis, the nanoUPLC was coupled to the mass spectrometer through a nano-electrospray ionization (nanoESI) source emitter.

### LC-QTOF-MS/MS analysis (DDA)

DDA (Data Dependent Analysis) analysis was performed on an SYNAPT G2-Si high-definition mass spectrometer (Waters) equipped with a NanoLockSpray dual electrospray ion source (Waters). Precut fused silica PicoTipR Emitters for nanoelectrospray, outer diameters: 360 μm; inner diameter: 20 μm; 10 μm tip; 2.5” length (Waters) were used for samples and Precut fused silica TicoTipR Emitters for nanoelectrospray, outer diameters: 360 nm; inner diameter: 20 μm; 2.5” length (Waters) were used for the lock mass.solution. The eluent was sprayed at a spray voltage of 2.8 kV with a sampling cone voltage of 25 V and a source offset of 30 V. The source temperature was set to 80 °C. The cone gas flow was 20 L/h with a nano flow gas pressure of 0.4 bar and the purge gas was turned off. The SYNAPT G2Si instrument was operated in DDA (data-dependent mode), automatically switching between MS and MS2. Full scan MS and MS2 spectra (m/z 400–2000) were acquired from 2 min after injection to 130 min in resolution mode (20,000 resolution FWHM at m/z 400) with a scan time of 0.1 s. Tandem mass spectra of up to ten precursors were generated in the trapping region of the ion mobility cell by using a collision energy ramp from 17/19 V (low mass, start/end) to up to 65/75 V (high mass, start/end). Charged ions (+2, +3, +4) are selected to be submitted to the MSMS fragmentation over the m/z range from 50 to 2000 with a scan time of 0.25 s. For the post-acquisition lock mass correction of the data in the MS method, the doubly charged monoisotopic ion of [Glu1]-fibrinopeptide B was used at 100 fmol/μl using the reference sprayer of the nanoESI source with a frequency of 30 s at 0.5μl/min into the mass spectrometer. ESI-QTOF were processed with MASCOT search engine (Matrix Sciences) using the protein sequences introduced in the Bacteria_Uniprot database.

### Relative catalytic efficiencies calculation

The relative catalytic efficiencies of a LarAH acting simultaneously on two substrates, S_X_ and S_Y_, at rates v_x_ and v_y_, were calculated using Equation (1),

(1) $$\frac{V_{X}}{V_{Y}}=\frac{\left(k_{cat}^{X}/K_{M}^{X}\right)\left[S_{X}\right]}{\left(k_{cat}^{Y}/K_{M}^{Y}\right)\left[S_{Y}\right]}$$

where if [S_X_] = [S_Y_] the [S] terms cancel, and the ratio of the *k*_cat_/*K*_M_ values for each substrate is equal to the relative rates at which the LarAH catalyzes the transformation of each substrate when both are present at equal concentrations with the enzyme in the same mixture (30). v_x_ and v_y_ were determined by quantifying the products P_X_ and P_Y_ either spectrophotometrically or by capillary electrophoresis. For a LarAH active on more than two substrates, we determined its catalytic efficiency for each of its substrates by testing combinations of substrate pairs with the LarAH.

### Phylogenetic analysis

The sequences of 12,441 LarAHs from the LarA superfamily (DUF2088) were retrieved from the Interpro entry IPR048068 on EMBL-EBI (accessed February 26, 2024) (21). This dataset of sequences was reduced by filtering all sequences by length with USEARCH v11.0.667 (command -sortbylength -minseqlength 300 -maxseqlength 600) and then by clustering all sequences using the UCLUST method implemented in the USEARCH package (command –cluster_fast –id 0.5 -centroids) (31), resulting in a list of 1212 representative LarAHs sequences. The 1212 LarAHs sequences were aligned with MAFFT v7.271 using FFT-NS-2 parameters (32, 33). The LarA superfamily phylogenetic tree was constructed from the alignment in the maximum likelihood framework using IQ-TREE v2.0.6 (34). Ten archaeal sequences from the DUF362 (Pfam entry PF04015) were used as outgroup since DUF362 and DUF2088 are part of the same clan (Pfam clan CL0471). The sequence evolution model, Q.pfam + R10 replacement matrix (35), was determined by ModelFinder as implemented in IQ-TREE2.0 (36). Branch supports were measured by ultrafast bootstrap approximation 2.0 conducted with 1000 replicates (option –bb 1000) (37). The tree was rooted with the outgroup and visualized using iTOL v5 (38).

## Data availability

Data are generally contained within the manuscript or the Supporting information file. Any data not contained herein are available from the authors by contacting Benoît Desguin by E-mail at benoit.desguin@uclouvain.be.

## Supporting information

This article contains supporting information (9, 10, 16, 39).

## Conflict of interest

The authors declare that they have no conflicts of interest with the contents of this article.
