# Supplementary material for: Unveiling 14 novel 2-hydroxy acid racemization and epimerization reactions in the lactate racemase superfamily
Source: J Biol Chem. 2024 Dec 10;301(1):108069. doi: 10.1016/j.jbc.2024.108069 (PMC11770544; doi:10.1016/j.jbc.2024.108069)
Supplement: Supporting information [file mmc1.docx]

**Supplementary material**

**Unveiling 14 novel 2-hydroxy acid racemization and epimerization reactions in the lactate racemase superfamily**

Julian Urdiain‑Arraiza^a^, Amandine Vandenberghe^a^, Gergana Dimitrova^a^ and Benoît Desguin^a,*^

**Content:**

**Figures S1-S7**

**Tables S1-S4**

**References**

**
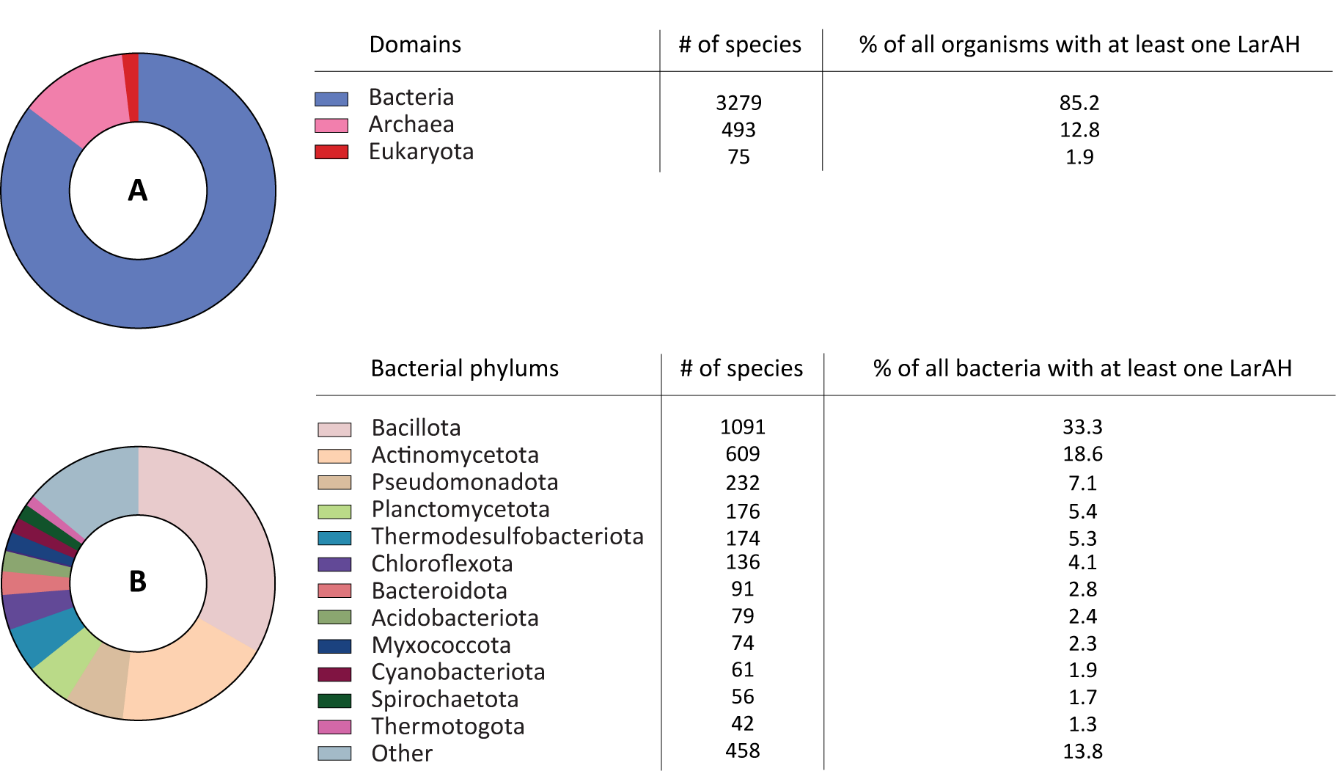
**

**Figure S1. Taxonomic distribution of LarAHs sequences.** (**A**) Taxonomic distribution of LarAHs sequences across domains. (**B**) Taxonomic distribution of LarAHs sequences within bacteria. The number of species represent the number of microorganisms in each domain/phylum that contain at least one LarAH. Data assembled from the Interpro entry IPR048068 on the European Molecular Biology Laboratory - European Bioinformatics Institute (EMBL-EBI) obtained on February 26, 2024.

**
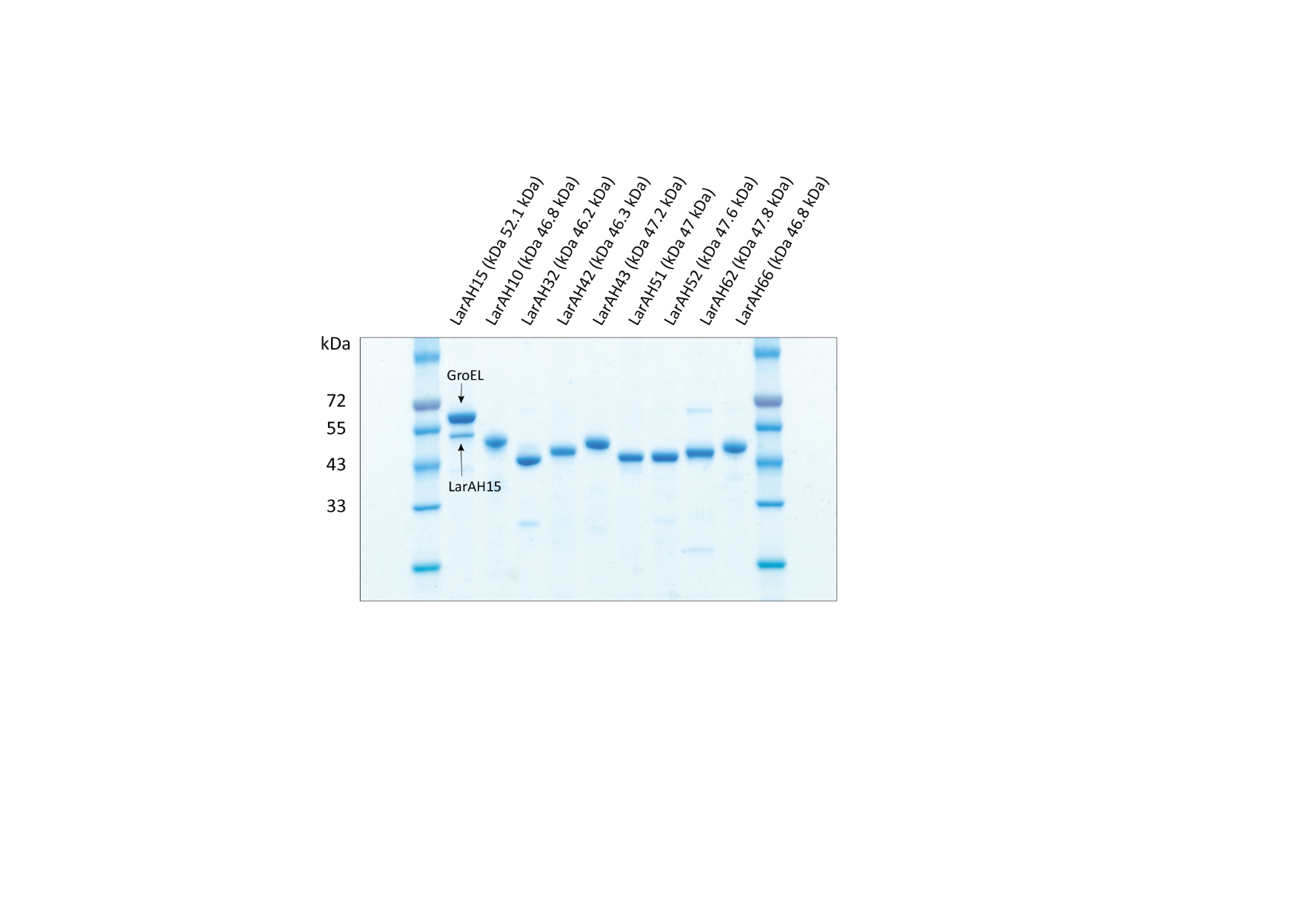
**

**Figure S2. Analysis of purified LarAHs from *E. coli* by SDS-PAGE.** The protein co-purified with LarAH15 was identified as the 60 kDa chaperonin protein Cpn60 (GroEL) by mass spectrometry analysis. LarA1 and LarAH5/Mar1 are not included, as their purification has been previously shown (10,9). Sequences of the proteins are provided in Table S3.


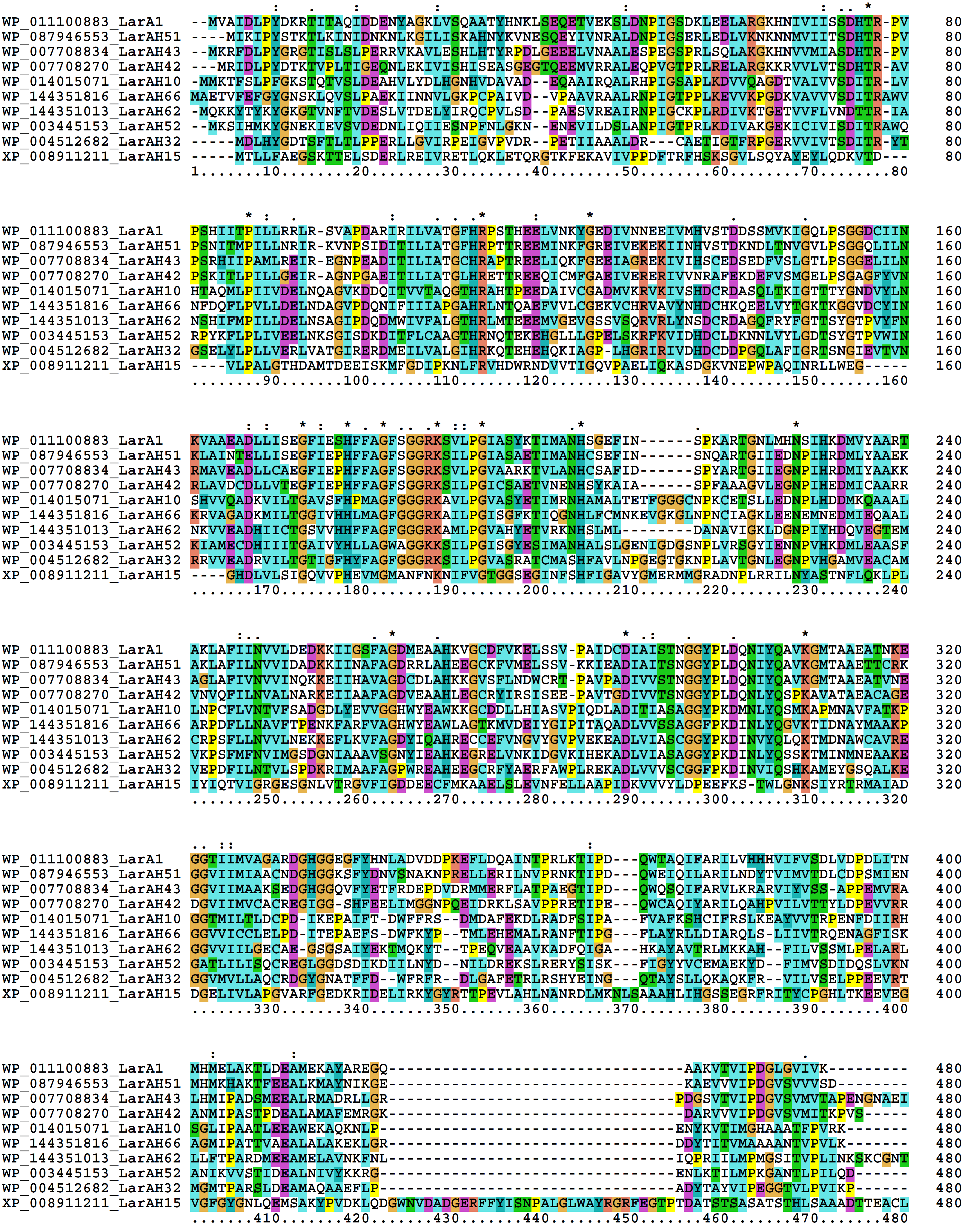
**
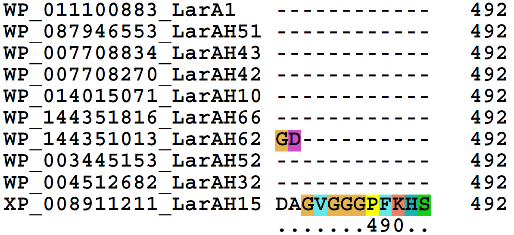
**

**Figure S3. ClustalX alignment of LarA and the 9 investigated LarAHs.** The residues conserved in all 10 sequences are indicated with an asterisk, the less conserved residues are indicated with one or two dots. The color scheme is the default colors of the ClustalX program.

**
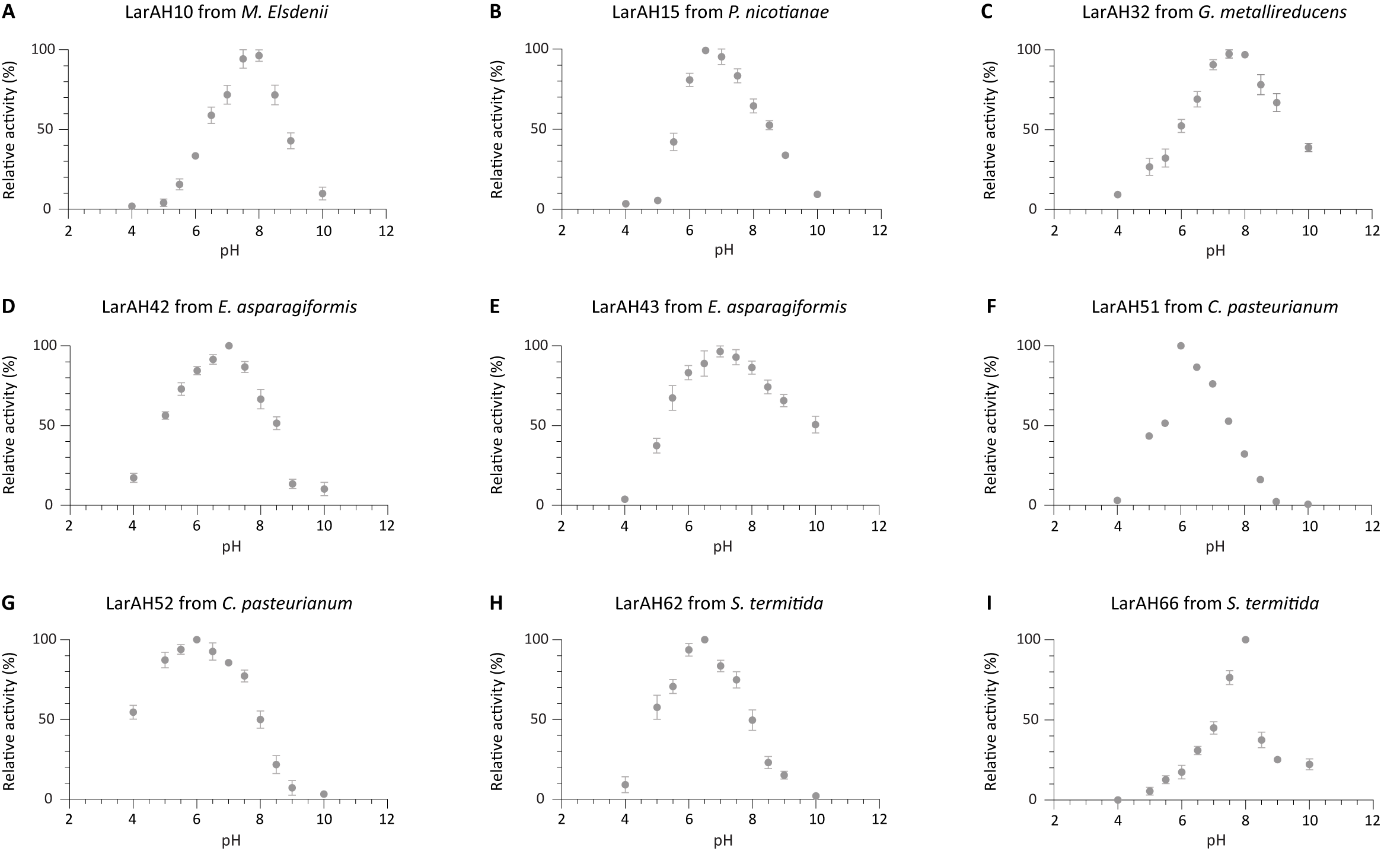
**

**Figure S4. pH-dependent activity of the 9 investigated LarAHs.** (**A**) Relative activity of LarAH10 from *M. Elsdenii* as a function of temperature, assessed for the C2-epimerization of D-mannonate. (**B**) Relative activity of LarAH15 from *P. nicotianae* as a function of temperature, assessed for the C2-epimerization of D-mannonate. (**C**) Relative activity of LarAH32 from *G. metallireducens* as a function of temperature, assessed for the racemization of L-2-hydroxyglutarate. (**D**) Relative activity of LarAH42 from *E. asparagiformis* as a function of temperature, assessed for the racemization of L-lactate. (**E**) Relative activity of LarAH43 from *E. asparagiformis* as a function of temperature, assessed for the racemization of L-lactate. (**F**) Relative activity of LarAH51 from *C. pasteurianum* as a function of temperature, assessed for the racemization of L-lactate. (**G**) Relative activity of LarAH52 from *C. pasteurianum* as a function of temperature, assessed for the racemization of D-2-hydroxy-4-oxo-phenylbutyrate. (**H**) Relative activity of LarAH62 from *S. termitida* as a function of temperature, assessed for the racemization of D-malate racemization. (**I**) Relative activity of LarAH66 from *S. termitida* as a function of temperature, assessed for the racemization of D-2-hydroxybutyrate. The error bars represent the standard error (n=3).

**
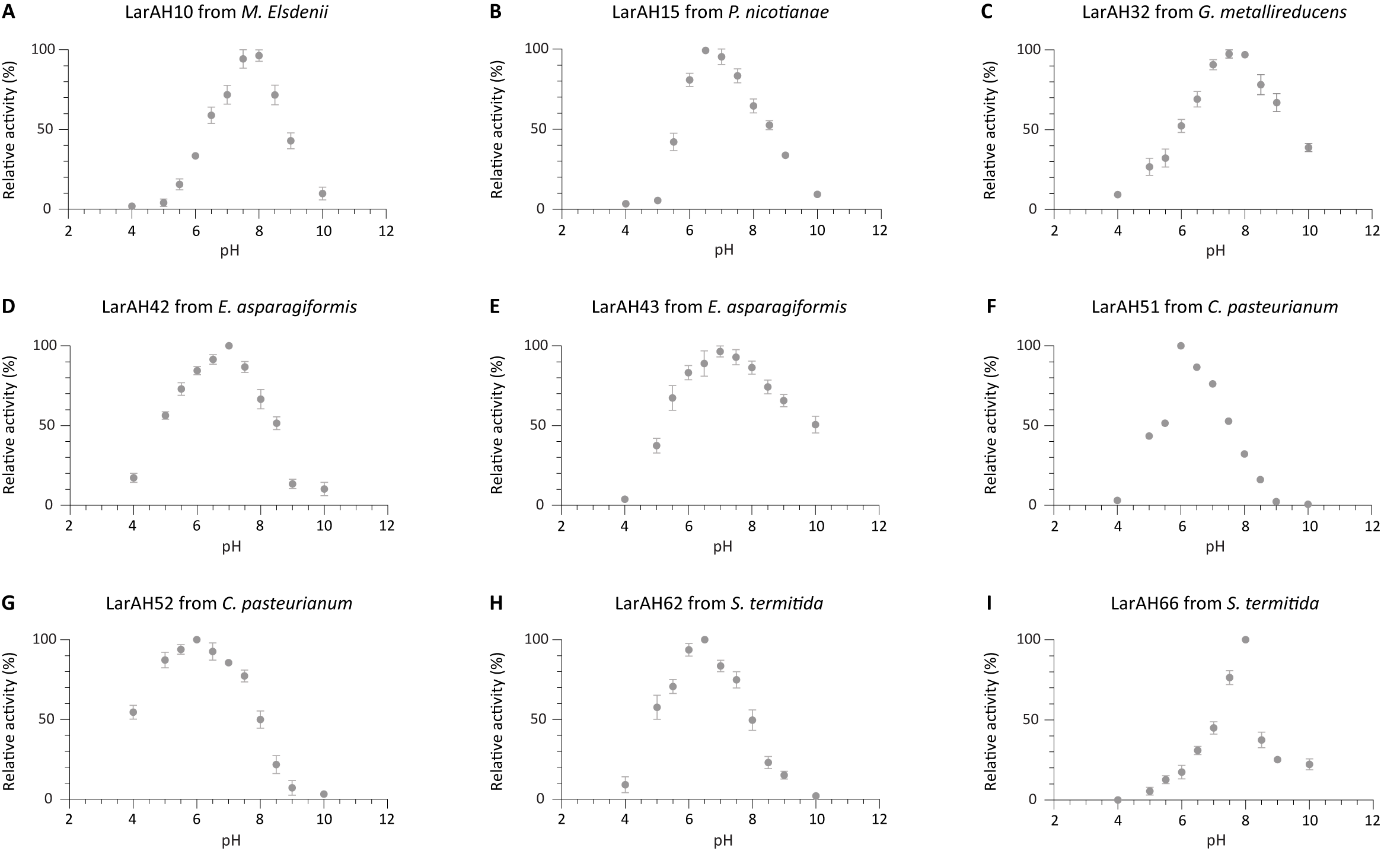
**

**Figure S5.** **Temperature-dependent activity of the 9 investigated LarAHs.** (**A**) Relative activity of LarAH10 from *M. Elsdenii* as a function of temperature, assessed for the C2-epimerization of D-mannonate. (**B**) Relative activity of LarAH15 from *P. nicotianae* as a function of temperature, assessed for the C2-epimerization of D-mannonate. (**C**) Relative activity of LarAH32 from *G. metallireducens* as a function of temperature, assessed for the racemization of L-2-hydroxyglutarate. (**D**) Relative activity of LarAH42 from *E. asparagiformis* as a function of temperature, assessed for the racemization of L-lactate. (**E**) Relative activity of LarAH43 from *E. asparagiformis* as a function of temperature, assessed for the racemization of L-lactate. (**F**) Relative activity of LarAH51 from *C. pasteurianum* as a function of temperature, assessed for the racemization of L-lactate. (**G**) Relative activity of LarAH52 from *C. pasteurianum* as a function of temperature, assessed for the racemization of D-2-hydroxy-4-oxo-phenylbutyrate. (**H**) Relative activity of LarAH62 from *S. termitida* as a function of temperature, assessed for the racemization of D-malate racemization. (**I**) Relative activity of LarAH66 from *S. termitida* as a function of temperature, assessed for the racemization of D-2-hydroxybutyrate. The error bars represent the standard error (n=3).

**
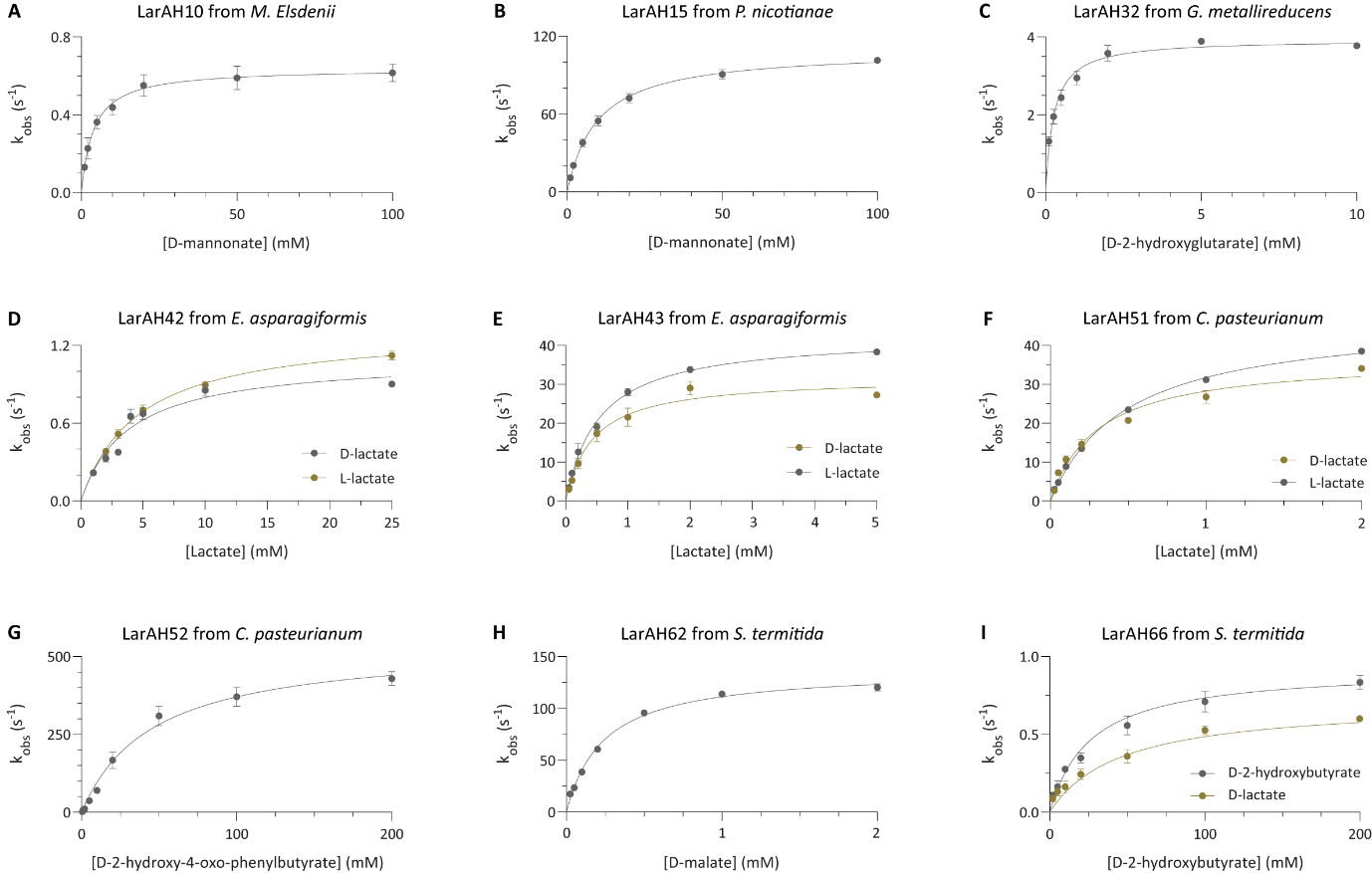
**

**Figure S6.** **Kinetic analysis of the 9 investigated LarAHs.** **(A**) D-gluconate/D-mannonate epimerization by LarAH10 from *M. Elsdenii*, (**B**) D-gluconate/D-mannonate epimerization by LarAH15 from *P. nicotianae*, (**C**) L-2-hydroxyglutarate racemization by LarAH32 from *G. metallireducens,* (**D**) D- and L-lactate racemization by LarAH42 from *E. asparagiformis,* (**E**) D- and L-lactate racemization by LarAH43 from *E. asparagiformis*, (**F**) D- and L-lactate racemization by LarAH51 from *C. pasteurianum,* (**G**) D-2-hydroxy-4-oxo-phenylbutyrate racemization by LarAH21 from *C. pasteurianum*, (**H**) D-malate racemization by LarAH62 from *S. termitida*, (**I**) D-2-hydroxybutyrate and D-lactate racemization by LarAH66 from *S. termitida*. The activities of the enzymes at the indicated substrate concentrations are shown as *k*_obs_ = *v*_0_/(E)_0_. The curves are the fitted curves using non-linear regression. The error bars represent the standard error (n=3).


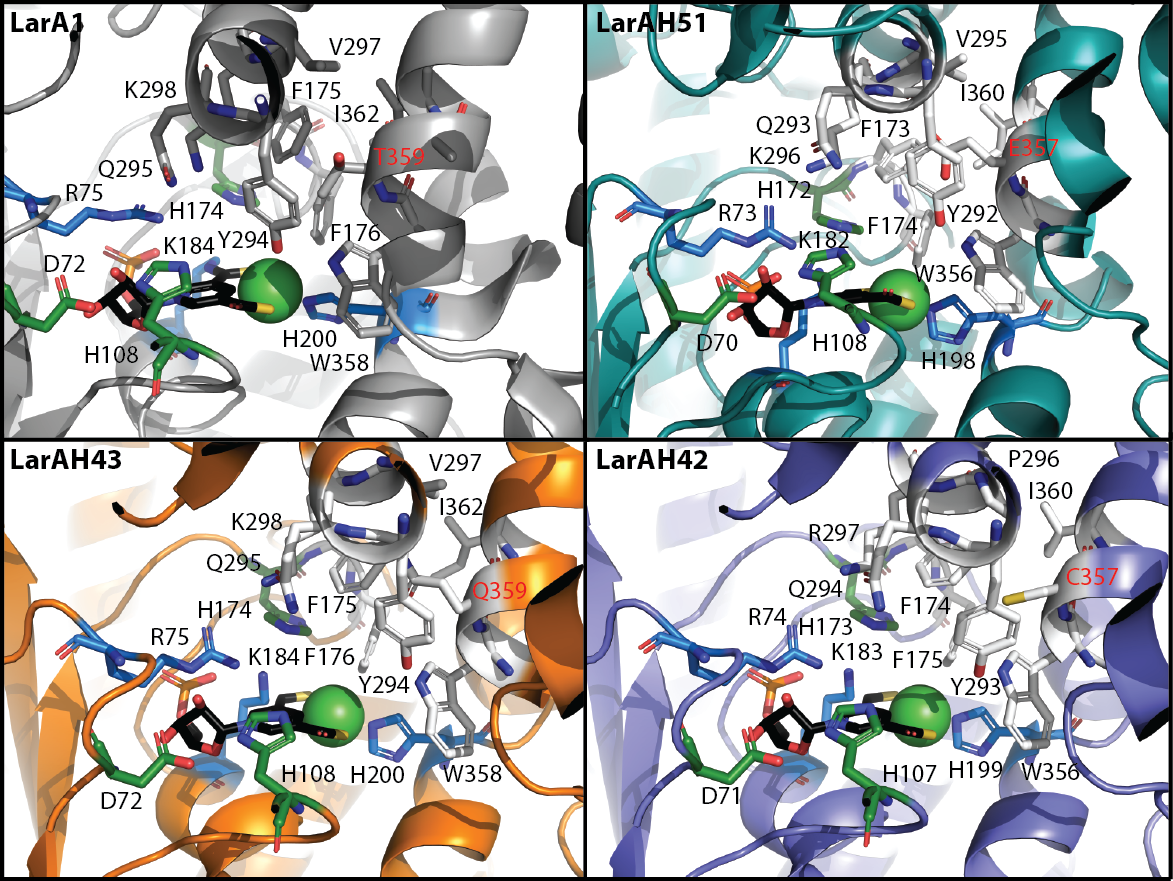


**Figure S7. Structure of LarA1 (PDB code 5HUQ) and structural models of 3 lactate racemases.** The catalytic residues are shown with their carbons in green, the residues important for NPN binding are shown with their carbons in blue, other residues probably important for substrate specificity are shown with their carbons in white. NPN carbons are in black. Oxygen atoms are in red, nitrogen atoms are in blue, sulfur atoms are in yellow, phosphate atoms are in orange, and nickel ion is shown as a green ball. The residues corresponding to T359 of LarA1 are labelled in red.

**Table S1. Prevalence of LarAH sequences among archaeal and bacterial taxa.** Data assembled from the database of Clusters of Orthologous Genes (COGs) entry COG3875 on the National Center for Biotechnology Information (NCBI) obtained on September 23, 2024.

| **Taxa** | **COG3875 data** | **% of organisms**  **with LarAHs** | | **Mean of LarAHs**  **per organism** | |  |
| --- | --- | --- | --- | --- | --- | --- |
|  |  |  | |  | |  |
| Archaea | [57/193 organisms 73 genes] | 29.5 | | 1.3 | |  |
| Asgardarchaeota | [7/8 organisms 20 genes] | 87.5 | | 2.8 | |  |
| Crenarchaeota | [0/29 organisms 0 genes] | 0 | | 0 | |  |
| Euryarchaeota | [36/103 organisms 38 genes] | 34.9 | | 1.1 | |  |
| Nitrososphaerota | [3/13 organisms 3 genes] | 23.1 | | 1 | |  |
| Thermoplasmatota | [7/17 organisms 7 genes] | 41.2 | | 1 | |  |
| Other Archaea | [4/23 organisms 5 genes] | 17.4 | | 1.2 | |  |
|  |  |  | |  | |  |
| Bacteria | [344/2103 organisms 550 genes] | 16.4 | | 1.6 | |  |
| Acidobacteriota | [11/16 organisms 14 genes] | 68.7 | | 1.3 | |  |
| Actinomycetota | [54/253 organisms 69 genes] | 21.3 | | 1.3 | |  |
| Aquificota | [0/9 organisms 0 genes] | 0 | | 0 | |  |
| Bacillota | [102/355 organisms 208 genes] | 28.7 | | 2 | |  |
| Bacilli | [11/152 organisms 11 genes] | 7.2 | | 1 | |  |
| Clostridia | [76/159 organisms 161 genes] | 47.8 | | 2.1 | |  |
| Erysipelotrichia | [1/14 organisms 1 genes] | 7.1 | | 1 | |  |
| Negativicutes | [10/12 organisms 29 genes] | 83.3 | | 2.9 | |  |
| Tissierella | [3/16 organisms 5 genes] | 18.7 | | 1.7 | |  |
| Other Bacillota | [1/2 organisms 1 genes] | 50 | | 1 | |  |
| Bacteroidota | [9/192 organisms 9 genes] | 4.7 | | 1 | |  |
| Bdellovibrionota | [0/7 organisms 0 genes] | 0 | | 0 | |  |
| Campylobacterota | [2/20 organisms 2 genes] | 10 | | 1 | |  |
| Chlamydiota | [0/12 organisms 0 genes] | 0 | | 0 | |  |
| Chlorobiota | [0/5 organisms 0 genes] | 0 | | 0 | |  |
| Chloroflexota | [8/19 organisms 14 genes] | 42.1 | | 1.7 | |  |
| Cyanobacteriota | [5/75 organisms 5 genes] | 6.6 | | 1 | |  |
| Defferibacterota | [7/7 organisms 7 genes] | 100 | | 1 | |  |
| Deinococcota | [1/7 organisms 1 genes] | 14.3 | | 1 | |  |
| Fusobacteriota | [1/9 organisms 1 genes] | 11.1 | | 1 | |  |
| Mycoplasmota | [2/24 organisms 2 genes] | 8.3 | | 1 | |  |
| Myxococcota | [15/17 organisms 21 genes] | 88.2 | | 1.4 | |  |
| Planctomycetota | [38/44 organisms 76 genes] | 86.4 | | 2 | |  |
| Pseudomonadota | [20/808 organisms 22 genes] | 2.5 | | 1.1 | |  |
| Alphaproteobacteria | [10/300 organisms 11 genes] | 3.3 | | 1.1 | |  |
| Betaproteobacteria | [3/166 organisms 3 genes] | 1.8 | | 1 | |  |
| Gammaproteobacteria | [6/338 organisms 7 genes] | 1.8 | | 1.2 | |  |
| Other Proteobacteria | [1/4 organisms 1 genes] | 25 | | 1 | |  |
| Spirochaetes | [7/22 organisms 14 genes] | 31.8 | | 2 | |  |
| Synergistetes | [6/10 organisms 13 genes] | 60 | | 2.2 | |  |
| Thermodesulfobacteriota | [27/56 organisms 45 genes] | 48.2 | | 1.7 | |  |
| Thermotogota | [6/12 organisms 6 genes] | 50 | | 1 | |  |
| Verrucomicrobiota | [3/24 organisms 7 genes] | 12.5 | | 2.3 | |  |
| Other bacteria | [17/100 organisms 20 genes] | 17 | | 1.2 | |  |
|  |  | |  | |  | |

**Table S2. LC-ESI-QTOF-MS analysis of the 9 investigated LarAHs.**

| **Match to*** | **Score** | **Sequence coverage (%)** |
| --- | --- | --- |
| LarAH10 | 658 | 96 |
| LarAH15 | 326 | 77 |
| LarAH32 | 588 | 67 |
| LarAH42 | 251 | 48 |
| LarAH43 | 3723 | 74 |
| LarAH51 | 3084 | 56 |
| LarAH52 | 4951 | 56 |
| LarAH62 | 15319 | 58 |
| LarAH66 | 3034 | 62 |

**Table S3. Bacterial strains and plasmids used in this study.**

| **Strains** | **Characteristic(s)** |  | | **Source or reference** |
| --- | --- | --- | --- | --- |
| *Lc. Lactis* NZ3900 | MG1363 derivative |  | | [39] |
| *E. coli* DH10B | F^-^ *endA*1 *recA*1 *galE*15 *galK*16 *nupG rpsL* Δ*lacX*74 Φ80*lacZ*ΔM15 *araD*139 Δ(*ara*,*leu*)7697 *mcrA* Δ(*mrr-hsdRMS*-*mcrBC*) λ^-^ | |  | Invitrogen |
| *E. coli* ArticExpress | Contains *Cpn60* and *Cpn10* from *Oleispira antarctica* | | | Agilent |
|  |  |  | |  |
| **Plasmids** |  |  | |  |
| pGIR012 | Cm^r^; pNZ8048 with DNA encoding the StrepII-tag sequence translationally fused to *larA* | LarA purification | | [10] |
| pGIR026 | Em^r^ Amp^r^; pGIR660 with DNA encoding the StrepII-tag sequence translationally fused at the 3ʹ-end of the *larB* ORF | LarB purification | | [10] |
| pGIR031 | Cm^r^; pNZ8048 with DNA encoding the StrepII-tag sequence translationally fused to *larC* | LarC purification | | [10] |
| pGIR076 | Amp^r^; pBADHisA with DNA encoding *larE* translationally fused to the StrepII-tag sequence | LarE purification | | [16] |
| pGIR313 | Amp^r^; pBADHisA with DNA encoding LarAH5/Mar1 | LarAHs purification | | [9] |
| pGIR315 | Amp^r^; pBADHisA with DNA encoding LarAH10 |  |  | [9] |
| pGIR321 | Amp^r^; pBADHisA with DNA encoding LarAH15 |  |  | This study |
| pGIR322 | Amp^r^; pBADHisA with DNA encoding LarAH32 |  |  | This study |
| pGIR323 | Amp^r^; pBADHisA with DNA encoding LarAH42 |  |  | This study |
| pGIR324 | Amp^r^; pBADHisA with DNA encoding LarAH43 |  |  | This study |
| pGIR325 | Amp^r^; pBADHisA with DNA encoding LarAH51 |  |  | This study |
| pGIR326 | Amp^r^; pBADHisA with DNA encoding LarAH52 |  |  | This study |
| pGIR327 | Amp^r^; pBADHisA with DNA encoding LarAH62 |  |  | This study |
| pGIR328 | Amp^r^; pBADHisA with DNA encoding LarAH66 |  |  | This study |

*Emr, Ampr, and Cmr indicate resistance to erythromycin, ampicillin, and chloramphenicol, respectively.

**Table S4. List of primers used in this study.**

| **Name** | **Sequence (5’-3’)** | **Reference/source** |
| --- | --- | --- |
| LarAH15_A | TTTCCATGGCAACCTTATTATTTGCGGAGG | This study |
| LarAH15_B | AAAGCTAGCTGAGTGTTTGAAAGGTC | This study |
| LarAH32_A | TTTCCATGGACCTGCACTACGGTG | This study |
| LarAH32_B | TTTGCTAGCCGGCTTAATAACCGGCAGCAC | This study |
| LarAH42_A | TTTACATGTCCAGAATTGACCTTCCCTACGACAC | This study |
| LarAH42_B | TTTGCTAGCGGATACCGGTTTTGTGATCATC | This study |
| LarAH43_A | CCCACATGTCTAAACGATTTGATTTACCCTACGG | This study |
| LarAH43_B | TTTGCTAGCTATTTCCGCGTTCCCGTTC | This study |
| LarAH51_A | TTTTACATGTCCATTAAAATTCCCTATTCAACAAAAACCC | This study |
| LarAH51_B | TTTGCTAGCATCACTGACAACCACTGATAC | This study |
| LarAH52_A | TTTTACATGTCCAAAAGTATTCATATGAAATATGG | This study |
| LarAH52_B | TTTGCTAGCATCTTGTAAAATGGGAAGTG | This study |
| LarAH62_A | TTTTACATGTCCCAAAAAAAATATACCTACAAGTATG | This study |
| LarAH62_B | TTTGCTAGCATCGCCGGTGTTG | This study |
| LarAH66_A | AAAACCATGGCGGAAACAGTATTTGAGTTTG | This study |
| LarAH66_B | TTTGCTAGCTTTCAATACCGGCACAGTAT | This study |
| pBAD_up | GCAACTCTCTACTGTTTCTCCATAC | This study |
| pBAD_rev | CCGCCAGGCAAATTCTG | This study |

† PciI, NcoI, NheI restriction sites introduced in the primers are underlined in primer sequences.
